# A case report of occult Cameron ulcer and a systematic review of the literature

**DOI:** 10.3389/fmed.2025.1677344

**Published:** 2025-09-17

**Authors:** Wei-Feng Li, Xiao-Ping Lei, Xi-Qiu Yu, Li Lv, Xiao-Dong Li

**Affiliations:** Department of Gastroenterology, Shenzhen Luohu People’s Hospital, Shenzhen, China

**Keywords:** Cameron ulcer, upper gastrointestinal bleeding, iron deficiency anemia, hiatal hernia, literature review

## Abstract

**Background:**

Cameron ulcer is a rare condition characterized by ulceration within a large hiatal hernia. Numerous studies have reported that Cameron ulcers are typically detected due to complications such as gastrointestinal bleeding or chronic iron deficiency anemia. However, diagnosis based solely on recurrent abdominal pain is exceedingly rare.

**Case presentation:**

A 46-year-old male presented repeatedly with unexplained abdominal pain over a four-year period. A definitive diagnosis of occult Cameron’s ulcer was established through a combination of gastroscopy, abdominal computed tomography (CT), and histopathological analysis. Following proton pump inhibitor (PPI) therapy, the patient’s symptoms showed significant improvement.

**Discussion:**

We report the first documented case of an occult Cameron ulcer presenting exclusively with recurrent abdominal pain. In addition, we conducted a comprehensive literature review of previously reported cases of Cameron ulcers. Clinicians should maintain a high index of suspicion for Cameron ulcer in middle-aged and elderly women with hiatal hernia who present with chronic iron deficiency anemia or upper gastrointestinal bleeding. For the diagnosis of occult Cameron ulcers, the integration of gastroscopy, abdominal CT, and histopathological analysis significantly enhances diagnostic accuracy. While the majority of Cameron ulcers respond well to medical management, including acid suppression therapy and iron supplementation, individualized surgical intervention should be considered for patients with severe or refractory complications.

**Conclusion:**

For patients presenting solely with abdominal pain and suspected Cameron ulcers, we recommend the integrated use of gastroscopy, abdominal CT, and histopathological examination to enhance diagnostic accuracy. In cases with associated complications, a tailored, individualized treatment strategy should be formulated based on clinical presentation and severity.

## Introduction

Cameron ulcer was first described by Cameron and Higgins in 1986 as ulcers occurring within the hernia sac of the esophageal hiatus (1). It is a rare type of ulcer, typically located at the level of the diaphragmatic impression. Epidemiological studies have indicated that the prevalence of Cameron ulcer among individuals with iron deficiency anemia or unexplained gastrointestinal bleeding ranges from 1.9 to 9.2%. In contrast, it is much less common in patients with overt gastrointestinal bleeding, with a reported prevalence of approximately 0.2% (2, 3). Most reports indicate that Cameron ulcers are typically diagnosed in the presence of concurrent iron deficiency anemia or upper gastrointestinal bleeding; diagnosis based solely on abdominal pain remains extremely challenging (4–18). This article presents a case of an occult Cameron ulcer that was difficult to diagnose. The distinguishing feature of this case is the presence of an ulcer located in the gastric body of the hernia sac in a patient without a history of iron deficiency anemia or gastrointestinal bleeding. The patient presented with intermittent abdominal pain, which improved following oral administration of a PPI. By reporting this case and reviewing the relevant literature, we aim to enhance the understanding, diagnosis, and management of Cameron ulcer.

## Case presentation

**Figure 1:**
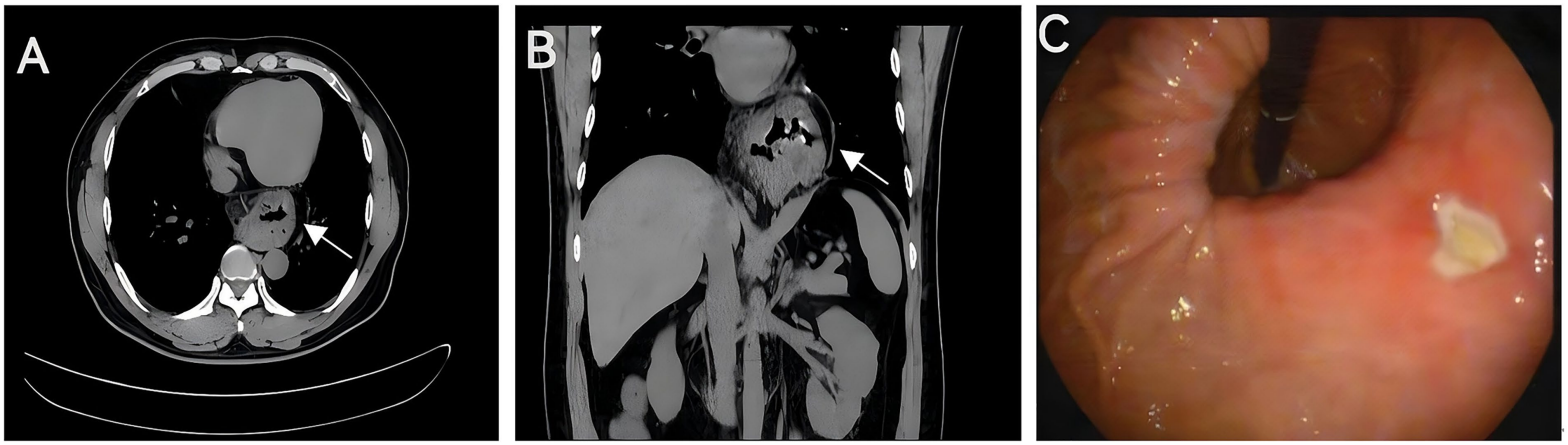
**(A)** Plain CT imaging demonstrates partial gastric herniation into the thoracic cavity, with the stomach positioned between the heart and abdominal aorta. **(B)** Coronal CT reconstruction more clearly delineates the herniation of gastric tissue into the thoracic cavity. **(C)** A large hiatal hernia is present, with a well-defined 5 mm × 6 mm ulcer observed along the lesser curvature of the upper gastric body, adjacent to the cardia.

**Figure 2:**
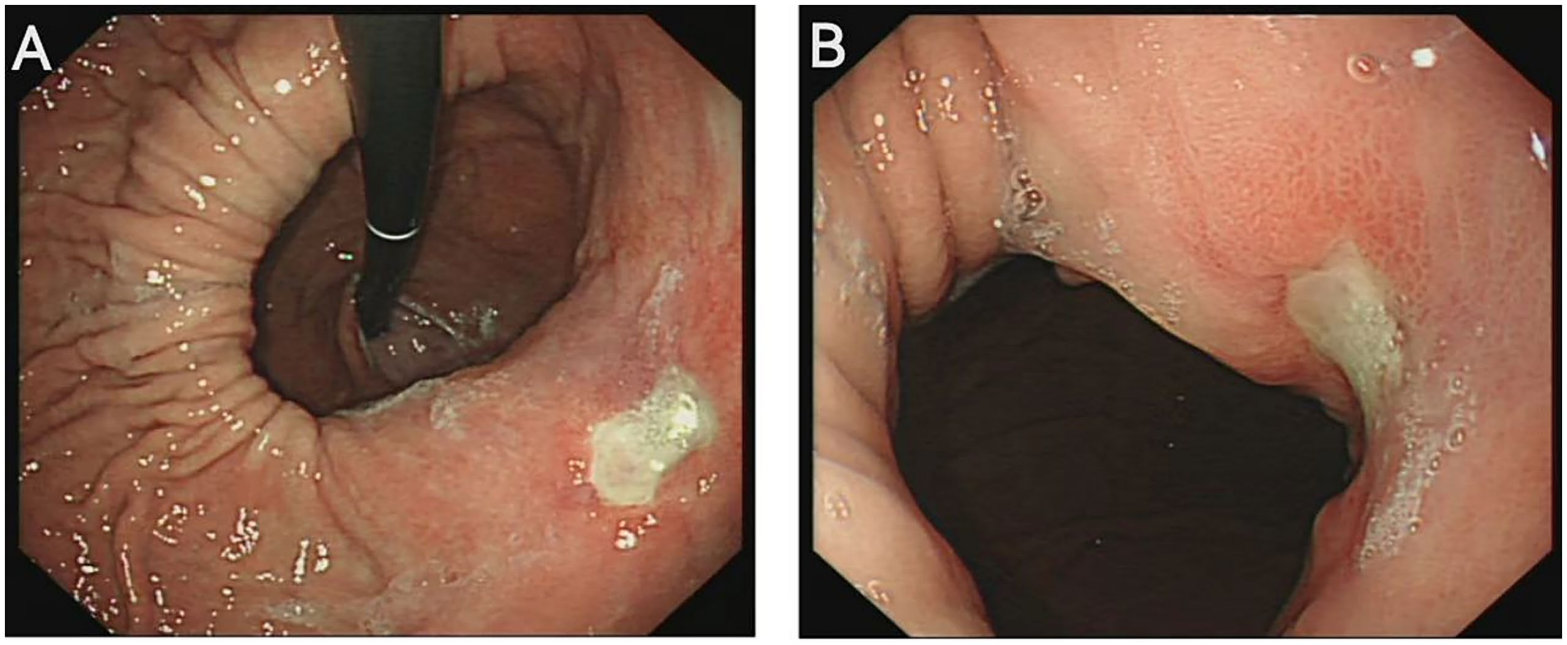
**(A)** Retroflexed endoscopic view reveals a large hiatal hernia, with a regular ulcer measuring 6 mm × 8 mm located along the lesser curvature of the gastric body. **(B)** Forward endoscopic visualization demonstrates that a portion of the stomach has formed a substantial hernia sac, within which Cameron ulcers are observed on the gastric body mucosa.

**Figure 3:**
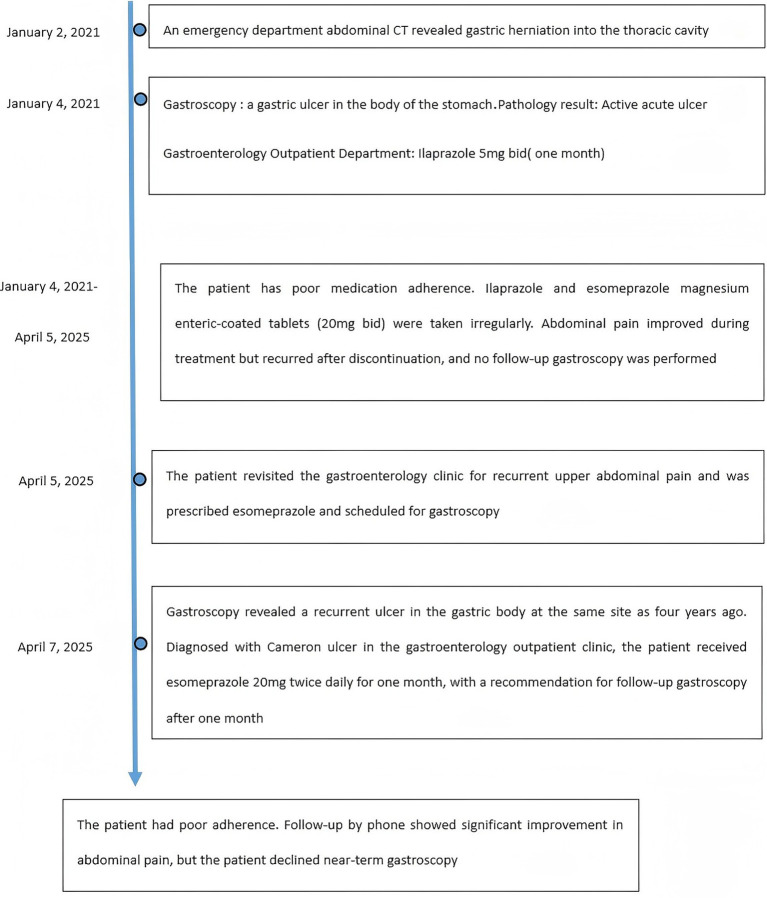
Time line of events.

A 46-year-old male presented to the gastroenterology outpatient clinic on April 7, 2025, with a 4-year history of intermittent upper abdominal pain and a recurrence lasting 1 day. Over the past 4 years, the patient experienced episodes of dull, upper abdominal pain characterized as paroxysmal, each lasting several minutes before spontaneous resolution. The pain was unrelated to meals, and there were no accompanying symptoms such as diarrhea, nausea, vomiting, chest tightness, chest pain, hematemesis, or melena. The patient reported having undergone a complete blood count in the past, with no significant abnormalities detected (no documentation available), and denied any prior history of anemia. Physical examination revealed no signs of anemia, including absence of conjunctival pallor. The abdomen was soft, with no tenderness or rebound tenderness noted upon palpation. On January 4, 2021, he visited our department due to persistent abdominal pain. As shown in Figure 1A (plain CT), the stomach had herniated into the thoracic cavity, located between the abdominal aorta and the heart. Figure 1B (coronal CT) further confirmed the presence of a hiatal hernia with gastric displacement into the thorax. Subsequently, gastroscopy was performed, and the findings are presented in Figure 1C. A large hiatal hernia was observed, with a regular ulcer measuring 5 mm × 6 mm located in the upper gastric body. The ulcer base is covered with a yellowish-white exudate, and the surrounding mucosa exhibits mild congestion. Four biopsy specimens were obtained and histopathological analysis revealed: (gastric body, biopsy) ulcer with acute inflammatory activity. Based on the available clinical and diagnostic findings, a benign ulcer was considered to be the most likely diagnosis. The patient was initially prescribed 5 mg of ilaprazole enteric-coated tablets twice daily for a maximum treatment duration of one month. Subsequently, the patient took the medication intermittently and irregularly. The patient also reported irregular use of esomeprazole magnesium enteric-coated tablets (20 mg twice daily) for a maximum continuous period of one month. Abdominal pain showed marked improvement during periods of medication adherence. However, the patient only resumed medication when symptoms recurred and remained off treatment during asymptomatic intervals. One day prior to the current visit (April 7, 2025), the patient experienced a recurrence of upper abdominal discomfort with identical characteristics. He subsequently underwent repeat gastroscopy, as shown in Figure 2. In retroflexed view (Figure 2A), an ulcer with similar location and morphology to that observed four years earlier was identified. In the forward view (Figure 2B), an ulcer was confirmed within the gastric body portion of the hiatal hernia sac. Biopsies were again taken from the ulcer margin, and histopathology revealed chronic active mucosal inflammation. The patient’s current physical examination findings are consistent with those recorded 4 years ago. Throughout the past 4 years, the patient has not experienced any symptoms suggestive of upper gastrointestinal bleeding or anemia-related dizziness. Integrating the available clinical data, laboratory results, and medical history, we conclude that this case represents an occult Cameron ulcer. The patient was prescribed esomeprazole magnesium enteric-coated tablets (20 mg) twice daily for a duration of 1 month. During the telephone follow-up, the patient reported a significant improvement in abdominal pain and the absence of other discomfort symptoms. A gastroscopy re-examination was recommended; however, due to poor patient compliance, the procedure has not yet been performed (Figure 3).

## Discussion

We present the first documented case of an occult Cameron ulcer. The patient did not report symptoms of anemia. Although a complete blood count was not available to definitively exclude anemia, the patient exhibited no signs of dizziness, showed no evidence of pallor or other physical manifestations of anemia, and had no history of upper gastrointestinal bleeding. The sole presenting symptom was recurrent abdominal pain. Based on these clinical findings, we conclude that this case represents an occult Cameron ulcer. A systematic literature search was performed on PubMed using “Cameron ulcer” as the key search term, with no restrictions on publication date. The initial search retrieved a total of 237 articles. After applying predefined inclusion and exclusion criteria—including only case reports and excluding reviews, clinical studies, and other non-primary reports—a final set of 15 case reports was selected for analysis, as summarized in Table 1. All of these cases were identified in the context of concurrent iron deficiency anemia or upper gastrointestinal bleeding (4–18). Therefore, we conducted a comprehensive review of the relevant literature. Analysis of the reported cases revealed a higher incidence among middle-aged and elderly individuals, with the youngest patient being 10 years old (7) and the oldest 88 years old (13). The condition appears to be more prevalent in women, with 11 female and 4 male cases reported. Among the cases, 8 were associated with iron deficiency anemia, 5 with upper gastrointestinal bleeding, and 2 with both complications. Based on this epidemiological analysis, middle-aged and elderly women with hiatal hernia who present with long-standing iron deficiency anemia or upper gastrointestinal bleeding should be considered at increased risk for Cameron ulcer.

**Table 1:** Summary of reviewed cases.

References	Sex	Age	Underlying disease	Symptoms	Iron deficiency anemia	Upper gastrointestinal bleeding	Diagnosis	Primary treatment	Outcome
Our case	Male	46	Not	Abdominal pain	The patient denies a history of anemia	Not	CT: a portion of the stomach has herniated into the thoracic cavity. Gastroscopy:an ulcer is located in the gastric body portion of the hernia sac.	PPI	Patient improved
Le et al. (4)	Female	37	Chronic gastritis and anemia	Abdominal pain and dizziness	Yes	Not	CT: a portion of the stomach has herniated into the thoracic cavity. Gastroscopy:an ulcer is located in the gastric body portion of the hernia sac.	BT + PPI + IS + surgery (hernia repair of the esophageal hiatus using a mesh patch, followed by Toupet fundoplication).	Patient improved and discharged
Singhai et al. (5)	Female	50	Not	Abdominal pain, constipation, and intermittent fever	Yes	Not	Gastroscopy: the ulcer is situated within the sac of the hiatal hernia.	BT + PPI + IS	Patient improved and discharged
Seid et al. (6)	Male	78	Gout-induced arthritis	Hematemesis and melena	Not	Yes	Gastroscopy: retroflexed view of the erosion with signs of recent bleeding	BT + PPI + surgery (unclear)	Patient improved and discharged
Silva et al. (7)	Male	10	Cerebral palsy 、 anemia and chronic pulmonary disease	Vomiting	Yes	Not	Gastroscopy: the ulcer is situated within the sac of the hiatal hernia.	Surgery: A laparoscopic herniorrhaphy and fundoplication were performed	The patient showed clinical improvement and was discharged from the hospital; however, he succumbed to respiratory failure 1 year later.
Iliev et al. (8)	Female	61	Post-stroke sequelae, anemia, and psoriasis vulgaris	Melena	Yes	Yes	Gastroscopy: a small macroscopically visible, not bleeding linear erosion within the hiatal hernia	PPI and a gel (containing hyaluronic acid, chondroitin sulfate, and poloxamer 407) for 3 months.	Patient improved and discharged
Romero et al. (9)	Female	66	Anxiety with personality disorder	Hematemesis and melena	Not	Yes	Gastroscopy:a large hiatal hernia with multiple linear erosions	PPI	Unclear
Mehershahi et al. (10)	Male	64	Hypertension, dyslipidaemia, gastroesophageal reflux and ischemic stroke	Hematemesis	Not	Yes	Gastroscopy:a large hiatal hernia with three Cameron ulcers. The largest lesion was of 10 mm with visible vessel but no active bleeding at its base.	EH + PPI + IS	Patient improved and discharged
Jabak et al. (11)	Male	80	Advanced Alzheimer’s disease, hypertension, and a history of transient ischemic attack	Hematemesis	Not	Yes	Gastroscopy:hiatal hernia with a 5-cm deep cratered ulcer at the diaphragmatic pinch. The ulcer base contained adherent clot and fibrinous material. Emergent angiography: a left gastric artery pseudoaneurysm located immediately adjacent to the ulcer.	Coil embolization of the pseudoaneurysm, PPI and surgery(A laparoscopic herniorrhaphy)	Patient improved and discharged
Bisogni et al. (12)	Male	72	Hypertension and benign prostatic hyperplasia	Hematemesis and melena	Not	Yes	CT, Gastroscopy: a large bleeding ulceration of about 5 cm size in diameter (Type Forrest 1A) located along a gastric mucosal fold at the diaphragmatic impression associated to a torsional hiatal hernia	BT + PPI + surgery (Minimally invasive atypical gastrectomy performed in conjunction with hiatal hernia repair)	Patient improved and discharged
Zullo et al. (13)	Female	66	Anemia	Fatigue	Yes	Not	Gastroscopy: the ulcer is situated within the sac of the hiatal hernia.	BT + PPI + IS	Patient improved and discharged
Zullo et al. (13)	Female	88	Anemia	Abdominal pain	Yes	Not	Gastroscopy: the ulcer is situated within the sac of the hiatal hernia.	PPI	Patient improved and discharged
Gupta et al. (14)	Female	51	Chronic obstructive pulmonary disease, emphysema, anemia, gastritis, gastroesophageal reflux disease, hiatal hernia, and Cameron lesions.	Fatigue, dizziness, coughing and fever	Yes	Not	Gastroscopy: the ulcer is situated within the sac of the hiatal hernia.	BT	Unclear
Maganty et al. (15)	Female	65	Coronary artery disease, chronic obstructive pulmonary disease, hypertension, diabetes, mild normocytic anemia, gastroesophageal reflux disease (GERD), dyslipidemia, depression, obesity, sleep apnea, and pulmonary fibrosis.	Hematemesis, several-hour history of weakness, nausea and sweating.	Yes	Yes	Gastroscopy: the ulcer is situated within the sac of the hiatal hernia.	PPI + IS	Patient improved and discharged
Jovanović et al. (16)	Female	56	Unclear	Unclear	Yes	Not	Gastroscopy: the ulcer is situated within the sac of the hiatal hernia.	PPI + IS + surgery (A laparoscopic herniorrhaphy and fundoplication were performed)	Patient improved and discharged
Chun et al. (17)	Female	71	Non-alcoholic steatohepatitis, hypertension and anemia	Dizziness, fatigue and melena	Yes	Not	Gastroscopy: the ulcer is situated within the sac of the hiatal hernia.	IS + PPI	Patient improved and discharged
Kapadia et al. (18)	Female	87	Atrial fibrillation	Hematemesis and melena	Not	Yes	Gastroscopy: the ulcer is situated within the sac of the hiatal hernia.	Unclear	Unclear

BT: Blood Transfusion; IS: Iron Supplementation; EH: Endoscopic Hemostasis.

Gastroscopy remains the gold standard for diagnosis. While most Cameron ulcers can be identified through gastroscopy alone, challenging cases may require the adjunctive use of abdominal CT and histopathological analysis to clarify the diagnosis. Katz et al. (19) proposed that diaphragmatic compression of vasculature may lead to ischemic changes in Cameron ulcers, offering a novel diagnostic perspective. For cases with diagnostic uncertainty, a combination of gastroscopy, abdominal CT, and pathology can facilitate accurate identification. However, the majority of current reports rely primarily on gastroscopy combined with abdominal CT for diagnosis. In conjunction with the challenging diagnostic case we reported, several difficulties were encountered during the diagnostic process. First, the patient presented solely with upper abdominal pain and exhibited no additional symptoms. A variety of conditions can manifest as upper abdominal pain, including acute pancreatitis, cholelithiasis, and malignant upper gastrointestinal tumors. To exclude these differential diagnoses, an abdominal CT scan was performed. Second, endoscopic examination revealed an ulcer located in the gastric body, which is atypical for Cameron ulcer, as this condition is most commonly identified near the cardia. The following differential diagnoses were considered: (1) Peptic ulcer associated with *Helicobacter pylori* (Hp) infection: These ulcers typically occur in the gastric angle or along the lesser curvature of the antrum, and are rarely found in the gastric body or hernia sac. Although the patient did not undergo a 13C-urea breath test, endoscopic findings based on the Kyoto classification—including the absence of chicken-skin mucosa, yellow pigmentation, mucosal congestion and redness, or turbid mucus in the gastric body, fundus, and antrum—suggested no evidence of Hp infection. Therefore, we concluded that the patient was likely Hp negative. (2) Signet ring cell carcinoma, cardia cancer, and other malignant tumors: These conditions typically present with irregularly shaped ulcers and surrounding stiff mucosa, which were not observed in this case. Endoscopic features and histopathological findings effectively ruled out malignancy. (3) NSAIDs-induced ulcers: These are commonly associated with a history of NSAIDs use and typically occur in the antrum, often presenting as multiple lesions. In contrast, the lesion in this case was solitary, and the patient had no history of NSAIDs use. Finally, histopathological analysis of the biopsy specimens did not reveal the typical ischemic changes characteristic of Cameron ulcer. This may be attributed to insufficient tissue sampling or superficial biopsy collection. In addition, Katz et al. (19) highlighted that variations in pathologists’ expertise may result in limited familiarity with Cameron ulcers, potentially compromising the accuracy of related diagnostic evaluations. Nevertheless, the histopathological findings definitively excluded malignancy. Taken together, the findings from abdominal CT, endoscopic evaluation, and histopathological analysis enabled a definitive diagnosis of Cameron ulcer.

Regarding treatment, it is essential to first understand the underlying pathogenesis. Numerous studies have reported potential mechanisms involved in the development of Cameron ulcers (2, 20). The currently accepted mainstream theory suggests that these ulcers result from mechanical trauma caused by persistent friction of mucosal folds at the level of diaphragmatic contraction. Additional proposed mechanisms include gastric acid-induced injury and localized ischemia due to diaphragmatic compression of the hernia sac (20). Based on these pathogenic mechanisms, the following treatment strategies are available:

1. Conservative pharmacological therapy: This approach involves the use of acid-suppressing agents and iron supplementation. PPIs and H2 receptor antagonists are most commonly prescribed to reduce gastric acidity (21–23). Iron supplements are administered for patients presenting with iron deficiency anemia. In the majority of previously documented cases, symptoms were effectively managed with PPIs and iron supplementation. Notably, Iliev (8) reported successful outcomes using a combination of hyaluronic acid, chondroitin sulfate, and PPI in the treatment of Cameron ulcers. For patients without life-threatening complications, conservative pharmacological therapy represents a viable and effective treatment option. This patient with a Cameron ulcer presents solely with abdominal pain, without concomitant severe anemia or gastrointestinal bleeding. PPI therapy has shown favorable efficacy. Therefore, we recommend continuing conservative management with PPI. A follow-up visit one month after initiation of treatment is advised, including a repeat gastroscopy to assess mucosal healing and therapeutic response. However, due to the patient’s poor adherence to medical recommendations, we plan to conduct subsequent follow-ups via telephone to monitor clinical progress and treatment compliance.
2. Surgical intervention: Studies indicate that the prevalence of Cameron lesions correlates with the size of hiatal hernias, with a disease risk of 10–20% when the hernia exceeds 5 cm in size (2). Hiatal hernia can not only cause esophageal-gastric mucosal laceration syndrome (24–26) and black esophagus (27), but also rare Cameron ulcers. Based on the underlying pathogenesis, surgical correction of large hiatal hernias may effectively treat Cameron ulcers. The most common types of hiatal hernia are the sliding and paraesophageal types, and the selection of surgical approach should be tailored according to hernia subtype (3, 28). A total of six case reports describe patients who underwent surgical intervention and were discharged following symptom improvement. Among these, three cases received laparoscopic hiatal hernia repair combined with fundoplication (4, 7, 16), one case with paraesophageal hernia underwent minimally invasive atypical gastrectomy in conjunction with hiatal hernia repair (12), one case underwent hiatal hernia repair alone (11), and the surgical procedure in the final case was not specified (6). Although there remains no definitive consensus regarding the choice between laparoscopic and open repair techniques or the necessity of concomitant fundoplication (2), available case evidence suggests that laparoscopic hiatal hernia repair with fundoplication is the most frequently employed surgical strategy for Cameron ulcers, particularly due to the high prevalence of sliding hiatal hernias (4, 7, 16). In this case of a concealed presentation, a significant portion of the stomach has herniated into the thoracic cavity through the esophageal hiatus, thereby warranting consideration of surgical intervention. The patient did not proceed with surgery because PPI therapy demonstrated clinical efficacy, and the patient exhibited suboptimal adherence to medical treatment, which contributed to their reluctance toward surgical management. We will continue to maintain close clinical follow-up to ensure timely reassessment and appropriate long-term care. In cases where Cameron ulcer is complicated by life-threatening massive gastrointestinal hemorrhage, the initial management should involve endoscopic hemostasis, which can be achieved through electrocoagulation of the bleeding vessel. If endoscopic hemostasis is ineffective, the subsequent intervention of choice is angiographic embolization, which controls bleeding by occluding the responsible vascular supply. Ultimately, surgical correction of hiatal hernia should only be considered after the patient’s hemodynamic status has been stabilized. In summary, for patients with Cameron ulcers and severe complications, surgeons should develop individualized treatment plans based on clinical presentation and patient-specific factors.

## Conclusion

Based on the findings from this case of occult Cameron ulcer and a comprehensive review of relevant literature, we recommend the integration of gastroscopy with abdominal CT and histopathological analysis as a diagnostic approach for occult Cameron ulcer. While most patients can achieve effective symptom relief through conservative pharmacological treatment, surgical intervention should be considered for those presenting with severe gastrointestinal bleeding or refractory iron deficiency anemia that is unresponsive to medical therapy.

## Data Availability

The original contributions presented in the study are included in the article/supplementary material, further inquiries can be directed to the corresponding authors.
